# Surface-Functionalizing Strategies for Multiplexed Molecular Biosensing: Developments Powered by Advancements in Nanotechnologies

**DOI:** 10.3390/nano14242014

**Published:** 2024-12-14

**Authors:** Shangjie Zou, Guangdun Peng, Zhiqiang Ma

**Affiliations:** 1 Center for Cell Lineage Technology and Engineering, Guangdong Provincial Key Laboratory of Stem Cell and Regenerative Medicine, Guangdong-Hong Kong Joint Laboratory for Stem Cell and Regenerative Medicine, GIBH-CUHK Joint Research Laboratory on Stem Cell and Regenerative Medicine, GIBH-HKU Guangdong-Hong Kong Stem Cell and Regenerative Medicine Research Centre, China-New Zealand Belt and Road Joint Laboratory on Biomedicine and Health, Guangzhou Institutes of Biomedicine and Health, Chinese Academy of Sciences, Guangzhou 510530, China; zou_shangjie@gibh.ac.cn; 2 Department of Biomedical Engineering, City University of Hong Kong, 83 Tat Chee Avenue, Kowloon, Hong Kong 999077, China

**Keywords:** surface functionalization, nanomaterials, biosensor, multiplexing, sensor performance

## Abstract

Multiplexed biosensing methods for simultaneously detecting multiple biomolecules are important for investigating biological mechanisms associated with physiological processes, developing applications in life sciences, and conducting medical tests. The development of biosensors, especially those advanced biosensors with multiplexing potentials, strongly depends on advancements in nanotechnologies, including the nano-coating of thin films, micro–nano 3D structures, and nanotags for signal generation. Surface functionalization is a critical process for biosensing applications, one which enables the immobilization of biological probes or other structures that assist in the capturing of biomolecules. During this functionalizing process, nanomaterials can either be the objects of surface modification or the materials used to modify other base surfaces. These surface-functionalizing strategies, involving the coordination of sensor structures and materials, as well as the associated modifying methods, are largely determinative in the performance of biosensing applications. This review introduces the current studies on biosensors with multiplexing potentials and focuses specifically on the roles of nanomaterials in the design and functionalization of these biosensors. A detailed description of the paradigms used for method selection has been set forth to assist understanding and accelerate the application of novel nanotechnologies in the development of biosensors.

## 1. Introduction

Biomolecules, including proteins, deoxyribonucleic acid (DNA), ribonucleic acid (RNA), and metabolites in the form of small molecules, are the bases for all kinds of biological activities. Biosensors, which aim at quantifying the levels of target biomolecules, are the basis for daily healthcare monitoring, diagnostics, food safety, and biomedical research.

Due to the limited representability of single biomolecules, multiplexing is crucial for the development of novel biosensing technologies. By enabling the simultaneous detection of multiple analytes from a single sample, multiplexed biosensors significantly enhance efficiency and reduce the time and resources required for testing. This capability is particularly important in clinical settings, where high-dimensional readouts provided by multiplexed biosensors provide more a comprehensive and accurate diagnosis, which can lead to timely treatment decisions, ultimately improving patient outcomes. Because of these advantages, the global market of multiplexed assay has reached USD 12.9 billion in 2023, and is predicted to reach USD 17.85 billion by 2025 [1]. Additionally, multiplexed biosensors facilitate comprehensive analysis, allowing for the identification of functional correlations between markers that may be overlooked when using traditional single-analyte approaches [2,3,4]. As a result, the development and applications of multiplexed biosensors hold great promise for enhancing our understanding of biological systems and addressing global health challenges.

Currently, the biosensors for molecular analytes can be roughly divided into several major categories. One major type of sensor is the electrochemical biosensor, which can be sub-divided into amperometric and voltammetric biosensors, targeting different types of analytes [5]. Apart from electrochemical biosensors, image-based biosensors (such as those based on colorimetric reactions and fluorescence) are also widely encountered due to their features of simple system design, low cost, and capability of generating high-dimension data. To achieve detection of analytes with a very low detection limit, optical biosensors such as Raman spectroscopy and surface plasmon resonance (SPR) are commonly applied [6].

One common process of measuring biomolecules includes three procedures: capturing molecules, signal amplification, and signal measurement. For different types of biosensors, nanomaterials are usually involved in different procedures and play different roles. For example, in electrochemical biosensors, nanomaterials are usually used for functionalizing electrodes, such as by modifying the electrode surface to facilitate the binding and capturing of biomolecules [7,8,9,10,11,12]. In contrast, in optical and image-based biosensors, nanomaterials with special quantifiable properties are usually functionalized by biological probes to serve as signal generators and provide quantifiable signals for measurement [13,14,15,16,17,18,19,20]. The selection of nanomaterials for biosensors, as well as the related surface-functionalizing strategies, are highly important for the performance of biosensors. Moreover, in actual applications, simultaneous quantification on multiple biomolecules is usually required, emphasizing the importance of nanomaterials and of functionalizing strategies that have good multiplexing potentials.

**Figure 1:**
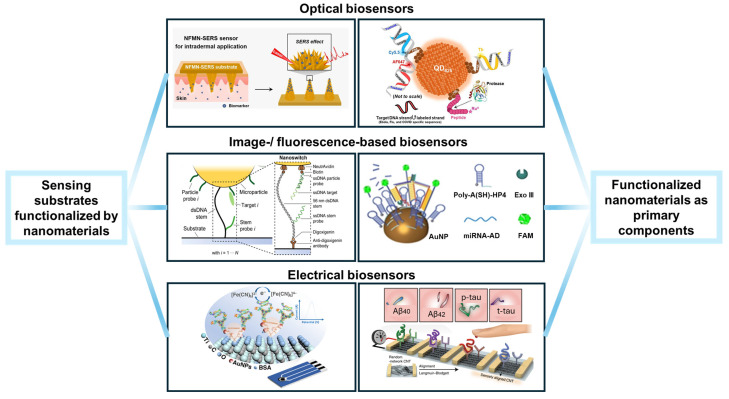
This schematic diagram demonstrates representative types of biosensors that involve nanomaterials in surface functionalization. Elements of the image are attributed as follows: Reproduced with permission from Ref. [21]. Copyright 2021, Elsevier. Reproduced with permission from Ref. [22]. Copyright 2021, American Chemistry Society. Reproduced with permission from Ref. [23]. Copyright 2022, Springer Nature. Reproduced with permission from Ref. [24]. Copyright 2024, American Chemistry Society. Reproduced with permission from Ref. [25]. Copyright 2023, Wiley. Reproduced with permission from Ref. [26]. Copyright 2020, Springer Nature.

This review serves as an overview of past studies related to biosensors, with emphases on nanomaterials’ functions and their roles in surface-functionalizing strategies, as illustrated in Figure 1. Aiming at bridging the development of novel nanomaterials and nanofabricating technologies and the instances of their applications in biosensors, we discuss and summarize the rationale behind the selections of materials and strategies. Representative examples of multiplexed biosensors were picked out to describe their special considerations and methods.

## 2. Surface Structures for Functionalization

Biosensors usually contain elements with largely different structures. The structural features of these elements are determined by their roles. In general, there are three types of surface structures: plane surfaces, 3D micro–nano structures, and nanoparticles.

### 2.1. Plane Surfaces

The plane surface is a fundamental structure in biosensors, exemplified by the electrodes used in electrochemical biosensors. These biosensors typically employ a three-electrode system, consisting of a working electrode, a counter electrode, and a reference electrode. To facilitate electrochemical reactions that generate current signals, molecular probes must be immobilized on the working-electrode surface [5].

Traditionally, electrodes of electrochemical biosensors are fabricated from metal materials such as gold, silver, or platinum, with gold being the most commonly used due to its excellent conductivity. In addition to its superior electrical properties, gold possesses the ability to form covalent gold–thiol bonds with biomolecules. Some studies have developed nucleic acid probes with terminal thiol groups. For instance, Sadat Mousavi et al. created a sensor array with electrodes functionalized with DNA probes via gold–thiol bonds, enabling the simultaneous measurement of five to ten genes [4]. The thiol group can also be easily conjugated to aptamers for immobilization on gold electrodes, allowing for the detection of a diverse range of analytes, including DNA sequences [27], proteins [28], exosomes [29], small-molecule metabolites [30], and antibiotics [31]. Furthermore, thiolated nanostructures can be immobilized on gold electrodes through gold–thiol bonds, enhancing sensor performance. This strategy has been shown to significantly improve hybridization efficiency and the signal-to-background ratio in miRNA level measurements [7].

**Figure 2:**
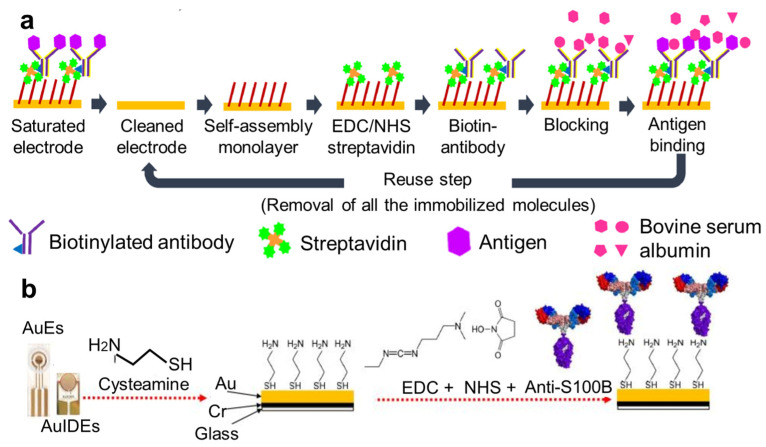
Functionalizing electrodes with self-assembled monolayer (SAM). (**a**) Functionalizing gold electrodes with a SAM based on 11-mercaptoundecanoic acid, which is regeneratable and contains carboxyl groups for connecting with streptavidin. Reproduced with permission from Ref. [9]. Copyright 2023, American Chemistry Society. (**b**) SAM on gold electrodes generated by cysteamine enables binding with carboxylated capturing probes. Reproduced with permission from Ref. [34]. Copyright 2021, Multidisciplinary Digital Publishing Institute.

In addition to the direct binding between thiolated probes and electrodes, gold–thiol bonds facilitate the formation of nanoscale self-assembled monolayers (SAMs). For example, Lee et al. utilized 11-mercaptoundecanoic acid to create SAMs on gold electrodes, which not only allowed for antibody binding through 1-ethyl-3-(3-dimethylaminopropyl) carbodiimide and N-hydroxysuccinimide (EDC-NHS) treatment but also enabled the regeneration of electrodes by washing away the SAM without damaging the gold surface [9] (Figure 2a). Gold–thiol bonds also support SAM formation using cysteamine and cystamine [32,33], both of which provide amino groups for binding with carboxyl groups via EDC-NHS treatment or with other amino groups through reactions with disuccinimidyl suberate, thereby facilitating the immobilization of capture probes or enzymes on electrode surfaces [34,35] (Figure 2b). Compared to the direct binding of probes to electrode surfaces, SAMs offer several advantages. In addition to facilitating the regeneration of sensing layers, as previously described [9], SAMs also reduce biofouling and enhance the stability of electrochemical biosensors by spatially separating the sensor surface from the environment [36]. SAMs with longer alkyl chains usually demonstrate better antifouling performance [37]. However, SAMs can also lead to baseline signal drift, which may increase noise levels [38]. Therefore, selecting appropriate molecules for SAMs and effectively managing their imperfections are crucial for maintaining the signal-to-noise ratio of the sensors.

Beyond gold–thiol bonds, gold surfaces can form strong bonds with selenium (Au-Se bonds). Some studies have employed selenocysteine to generate selenocysteine SAMs [39] which contain carboxyl terminals that can be coupled with an EDC-NHS treatment for immobilizing molecules within an amino group. Notably, this method can be used to bind streptavidin (SA) on gold electrode surfaces [39]. The SA–biotin bond, known for its strength, is commonly used to immobilize molecular probes on electrodes [39,40]. Simpler methods, such as direct physical adsorption, are also available for functionalizing gold surfaces with SA [41]. However, covalent and SA–biotin bonds are generally more robust and stable than physical adsorption. While the SA–biotin bond is known for its high stability, challenges arise from the structural characteristics of SA. As a tetramer, SA can complicate the control of probe orientation [42], potentially impacting the reproducibility and sensitivity of biosensors. Additionally, the ability of multiple biotinylated probes to bind to a single SA molecule may introduce steric hindrance, which can further hinder the detection of analytes.

Recently, researchers have begun exploring novel materials like graphene for electrode fabrication due to the exceptional electrical properties of these materials. However, the surface-functionalization strategies for these materials differ from those used for metals. Physical adsorption is frequently employed to immobilize traditional capture probes on graphene or carbon electrodes. For instance, antibodies can be immobilized on carbon electrodes through hydrophobic interactions [43]. Additionally, SA can be physically adsorbed onto graphene electrodes, sometimes aided by electrode polarization, allowing for the immobilization of antibodies or aptamers via SA–biotin binding [44,45]. To achieve covalent binding for the immobilization of antibodies or amine-conjugated DNA probes, graphene may need to be pre-modified with 1-pyrenebutanoic acid succinimidyl ester to introduce a succinimide group for connection with amine groups [46], or by introducing carboxyl groups through ultraviolet–ozone treatment [47] or anodizing graphene oxide with NaOH [48]. Notably, carboxyl functionalization using ultraviolet–ozone has been successfully applied to functionalize sensor arrays made from carbon nanotubes for antibody binding in the diagnosis of Alzheimer’s disease [26].

While biological probes can be directly immobilized on electrodes made from novel materials like graphene, the functionalization process is complex. Additionally, binding efficiency is often limited by the surface area and inherent properties of the electrode materials. Another common approach used to assist in the binding of probes is depositing extra nanomaterials in order to facilitate probe immobilization [49,50]. For instance, a nanolayer of gold coating is frequently used to functionalize electrodes made from materials with a low affinity for biological probes, such as carbon, silver, and innovative nanomaterials like MXene [51,52]. This strategy leverages the strengths of each material to achieve synergistic effects. Furthermore, materials with a high affinity for specific analytes can be deposited onto electrodes to create adsorptive structures, such as porous films. These structures are particularly effective for detecting small molecules [53].

Nanoparticles are also widely used as a material to modify electrodes. One important reason for the use of nanoparticle deposition on electrodes is to make use of its function of providing a high surface area for high-density probe binding. One common choice is gold nanoparticle (AuNP), which allows efficient binding of probes or SAM molecules with thiol groups [54,55]. Pt nanoparticles can also be applied to modify the electrode surface, facilitating the binding of probes through Pt-S bonds [56]. Beyond merely increasing surface area, nanoparticles impart valuable properties that enhance sensing mechanisms. For instance, lead sulfide colloidal quantum dots (QDs) can be deposited on gold electrodes, leveraging their surface ligand exchangeability to adsorb antigens for antibody detection [57]. Graphene QDs can function as nanozymes, catalyzing electrochemical reactions and enabling the development of enzyme-free systems for protein detection [58]. Overall, the integration of nanoparticles into electrode design not only improves sensitivity but also broadens the scope of applications in biosensing technologies.

Molecularly imprinted polymer (MIP) is another promising functionalizing method that does not rely on chemical binding. This technique creates a nanolayer of polymer on the surface of electrodes, one designed with specific affinities for target analytes, enabling effective capture and recognition. A significant advantage of MIPs is their adaptability: they can be customized for different target analytes and can be applied to a diverse array of electrodes, including graphene and glassy carbon. This flexibility allows for the detection of a wide range of molecules, extending far beyond just proteins and nucleic acids [10,11,12,59]. Additionally, MIP layers exhibit greater stability compared to traditional probes, making them suitable for applications in complex and extreme environments [60]. Despite their broad adaptability, MIP remains relatively underutilized as a surface-functionalization technique for biosensors. This is primarily due to significant challenges in its fabrication process, including difficulties in template removal (which can greatly impact target binding properties), selection of appropriate monomers, and management of environmental variables such as pH in the synthesis of MIP [61].

In recent years, advancements in chemical methodologies have broadened the range of probe-binding techniques available. Among these, click chemistry has emerged as a powerful approach, featuring highly efficient and selective reactions that facilitate the rapid assembly of molecules. A key functional group in click chemistry is the azide group (-N_3_), which can react with alkynes (carbon-carbon triple bonds) to form 1,2,3-triazoles. Building on this reaction, Xie et al. developed an aptasensor for lysozymes by modifying the surface of AuNP-coated carbon electrodes with 10-azidoundecan-1-thiol. This modification generates a SAM with azide terminals, enabling the binding of alkyne-modified aptamers [55]. Compared with traditional methods for binding biological probes (such as direct binding to the electrode’s surface through gold–thiol bond or EDC/NHS-mediated binding between carboxyl and amino group), this click chemistry, assisted by SAM, has demonstrated superiority as a technique for optimizing surface properties and achieving the higher binding efficiency of probes [55]. Furthermore, the use of click chemistry for assembly can effectively preserve the advantageous properties of surface modifications, such as antifouling characteristics, during the binding of probes. This preservation enhances the overall sensing performance [62]. While click chemistry has not yet gained widespread adoption in the surface functionalization of biosensors (mainly due to the cost and complexity involved in preparing electrodes and probes with the necessary functional groups), its strong compatibility and synergistic potential with SAM suggests that it will play a significant role in future advancements.

In addition to the advantages and disadvantages of the various surface-functionalization strategies discussed above, the reusability of biosensors is an important consideration that is often overlooked. Generally, covalent bonding of probes to the electrode surface enhances long-term usability. Among currently available biosensors utilizing covalent binding, gold is the most widely used electrode material. However, graphene- and carbon-based electrodes are believed to offer better stability and reusability compared to gold [9,63,64]. While SAMs show promise for improving the reusability of gold electrodes, selecting and fabricating an appropriate SAM can be challenging. Longer SAMs may compromise electron transport, whereas shorter SAMs tend to be less stable [64]. Therefore, developing functionalization methodologies that utilize stable electrode materials, ensure robust probe binding, and maintain good electrical properties is crucial for the practical application of biosensors. Notably, innovations in materials may help address the limitations of current methodologies. For instance, Wan et al. developed an electrochemical biosensor for pesticides by introducing polymeric ionic liquids onto a glassy carbon electrode. This approach provides a stable environment for immobilizing and encapsulating gold nanoparticles (AuNPs) on the sensor surface while allowing for a modifiable interface that facilitates the incorporation of additional components with favorable electron transport properties [65]. Surface modification using composite nanomaterials may represent a promising strategy for leveraging the advantages of different materials and functionalization methods.

In summary, the functionalization of planar electrodes primarily involves the binding or attachment of biological probes, often facilitated by a nanolayer coating known as a SAM. Electrodes can also be constructed from or coated with nanomaterials to enhance biomolecule binding or improve electrical properties. The high flexibility of functionalization allows for the easy construction of multiplexed biosensors by integrating multiple electrode systems, each functionalized with different probes [8,26,48].

### 2.2. 3D Structures

3D micro–nano structures are commonly utilized in biosensors for two primary purposes: signal amplification and analyte sampling.

**Figure 3:**
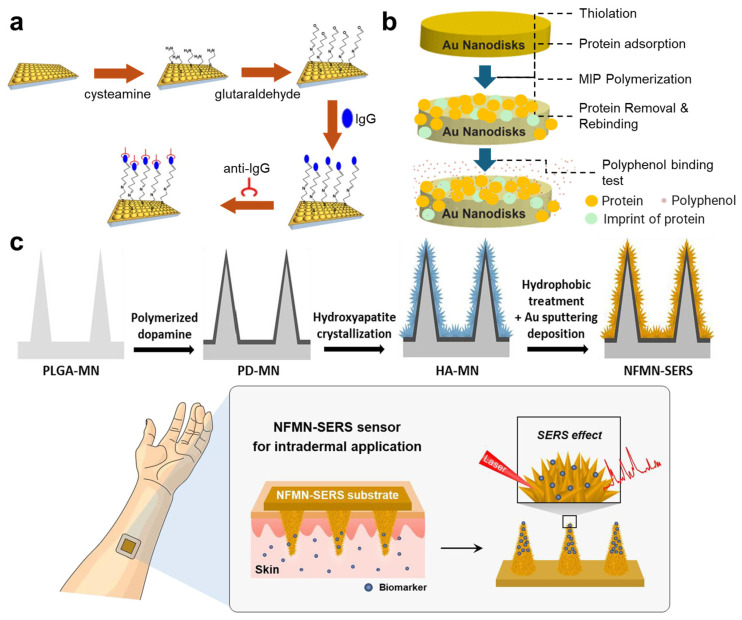
Functionalization of 3D biosensing substrates. (**a**) SAMs on gold electrodes generated by cysteamine conjugated with capturing probes with amino groups through the mediation of glutaraldehyde. Reproduced with permission from Ref. [69]. Copyright 2017, Springer Nature. (**b**) Generating molecularly imprinted polymer layers on gold nanodisks for measuring protein–polyphenol interactions based on surface plasmonic resonance. Reproduced with permission from Ref. [70]. Copyright 2016, American Chemistry Society. (**c**) Decorating hydroxyapatite nanoflowers on poly(lactic-co-glycolic acid) microneedles for intradermal sampling and detection of small molecules by surface-enhanced Raman spectroscopy. Reproduced with permission from Ref. [21]. Copyright 2021, Elsevier.

Signal amplification through 3D nanostructures is essential for optical biosensors, particularly in surface-enhanced Raman spectroscopy (SERS) and surface plasmonic sensors. In SERS biosensors, these nanostructures play a crucial role in enhancing Raman signals, thereby improving the sensitivity and accuracy of the detection process [66,67]. Likewise, sensors that utilize SPR also employ 3D nanostructures to enhance the coupling conditions between an incident light beam and the surface plasmons [68,69] (Figure 3a). Most of these sensors are functionalized using techniques commonly employed in the fabrication of electrochemical biosensors, such as the formation of gold–thiol bonds [66,67,68] and Au-Se bonds [69] between probes and a gold-based surface. In addition to direct binding, MIPs are also valuable in optical biosensors. Guerreiro et al. demonstrated the application of thiophenecarboxylic acid to create a thiol layer on gold nanodisks, followed by the generation of MIPs through protein adsorption, polymerization (using methacrylic acid and (vinylbenzyl)trimethylammonium chloride), and subsequent protein removal [70] (Figure 3b). The resulting nanocomplex can rebind with proteins, allowing for the evaluation of a protein’s affinity to various polyphenols, based on SPR measurements [70] (Figure 3b).

3D nanostructures are also prominent in electrochemical biosensors. Kim et al. developed a woodpile-like platinum (Pt) nanowire array that significantly enhanced the electrochemically active surface area, facilitating efficient charge transfer for the ultrasensitive detection of axonal neuron-specific proteins associated with Alzheimer’s disease [71]. Notably, they created a SAM using mercaptoundecanoic acid and mercaptohexanol, followed by the immobilization of antibodies through carbodiimide-assisted covalent binding [71].

In addition to signal modulation, 3D structures can be tailored for specific sampling requirements. For instance, microneedles have been utilized in various studies to facilitate sampling from tissues at specific depths. Researchers have integrated microneedles into biosensor systems using two primary approaches. The first approach involves using microneedles solely for sampling, with sensing electrodes connected to them for signal detection [31,72,73]. This design does not necessitate direct modification of the microneedle surfaces for molecular capture or signal generation. The second approach employs microneedles as the site for signal detection, which requires surface functionalization directly on the microneedles. One example of this is the use of gold (or gold-coated) microneedles functionalized with aptamers via gold–thiol bonds, enabling the monitoring of multiple molecular biomarkers in interstitial fluid through real-time electrochemical detection [74,75,76]. In these studies, microneedle arrays were incorporated into a three-electrode system, with aptamers immobilized on the microneedles featuring a redox reporter at their distal end, away from the sensor surface [74,75,76]. Upon capturing the target analytes, the aptamer undergoes a conformational change that brings the redox reporter closer to the sensor surface, affecting electron transfer and generating a change in the square-wave voltammogram peak current, which can be measured through voltammetric interrogation. This functionalization and sensing mechanism allows for straightforward upgrades to multiplexing by physically dividing the microneedle arrays into multiple sections separated by walls to enable independent aptamer deposition and target detection [74]. Notably, these studies also modified the microneedle surface with a self-assembled monolayer (SAM) formed by alkylthiols, which not only provides binding sites for aptamers but also stabilizes the electrodes against fluctuations in capacitance [74,75,76,77]. Furthermore, microneedles with nanostructures can also enhance optical biosensors. For example, a poly(lactic-co-glycolic acid) microneedle modified with polydopamine and decorated with hydroxyapatite nanoflowers has been employed for intradermal sampling and the detection of small molecules using SERS [21] (Figure 3c).

Apart from signal modulation and sampling, a major advantage of 3D micro–nanostructures is their ability to achieve a high probe density, which is particularly beneficial for sensors that require ultrasensitive detection of analytes. For instance, polystyrene microbeads have been incorporated onto the surfaces of carbon electrodes, significantly increasing the surface area available for loading aptamers through direct incubation in a simple buffer solution [33]. Similarly, the surfaces of gold electrodes can be roughened using electrical pulses to create 3D nanostructures, facilitating the binding of a greater number of aptamers [78]. Indeed, enhancing surface roughness by generating 3D nanostructures is a common strategy used to support high-density immobilization of probes and biomolecules [78,79,80]. However, it is important to note that random and uncontrolled surface roughness can negatively impact the stability and reproducibility of sensors [81,82]. To mitigate the adverse effects of nanoscale defects, various methods can be employed to refine the sensor surface, including electropolishing [83], SAM [81], annealing [84], and electrodeposition [85]. Overall, optimizing the surface properties of 3D micro–nanostructures is crucial for enhancing the performance of electrochemical sensors.

In contrast to electrical and optical biosensors, which are often constrained by the physical properties of specific materials, fluorescence- and image-based biosensors offer greater flexibility in material selection. This versatility allows for a wider array of strategies for surface functionalization. For instance, diamond microneedles on a silicon base functionalized with aptamers or RNA-binding proteins have been employed to detect intracellular proteins and microRNAs through fluorescent signals in a technique known as “molecular fishing” [86,87]. To modify diamond microneedles with biological probes, the two studies treated the device with piranha solution to introduce hydroxyl groups on the silicon surface; this was followed by the application of (3-aminopropyl)triethoxysilane to create amino tails that serve as binding sites for biological probes [86,87]. Multiplexing on these microneedles is typically achieved using signaling probes labeled with different fluorescent markers. For example, Wang et al. functionalized silicon microneedles with the universal double-stranded RNA binding protein p19 [87]. “Bait” RNA sequences were pre-delivered intracellularly and hybridized with target microRNAs to form double-stranded structures that can be captured by the p19 protein probe. After the capturing of the target, the microneedle array was retrieved; this was followed by a hybridization chain reaction to generate and amplify fluorescent signals for quantifying microRNAs, with different fluorescence indicating different microRNAs [87]. Additionally, methacrylated hyaluronic acid (MeHA) hydrogel-based microneedles have been utilized for the rapid measurement of glucose, adenosine triphosphate, l-tyrosinamide, and thrombin [88]. In one study, aptamer probes labeled with fluorophore were immobilized on the microneedles through covalent bonding between hyaluronic acid and acrydite groups, facilitated by a photoinitiator and ultraviolet exposure [88]. The binding of target analytes results in the detachment of the displacement strand-quencher from the aptamers, leading to fluorescent emission [88]. In some instances, the hydrogel coating can also function as a means of surface functionalization, which is useful in sampling from tissues. For example, one study introduced an alginate-coated poly(l-lactide) microneedle with alkyne-modified peptide nucleic acid probes embedded in the alginate hydrogel layer for sampling from interstitial fluid [89]. This microneedle can be functionalized with various peptide nucleic acid sequences that are complementary to different cell-free nucleic acid biomarkers, enabling sampling for subsequent in vitro multiplexed analysis [89]. Moreover, polymers can also be utilized in the fabrication of microneedles. For instance, polystyrene can effectively attract and immobilize proteins, such as antibodies, on its surface through hydrophobic interactions with nonpolar residues on the proteins, making it suitable for biomarker detection [90].

In summary, 3D micro–nano structures significantly boost the sensitivity and applicability of biosensors by improving signal amplification and analyte sampling, as well as increasing the active surface area for probe binding and efficient charge transfer. A diverse array of materials can be utilized in the development of biosensors featuring 3D micro–nano structures, including metals, hydrogels, polymers, and silicon. It is essential to carefully select the appropriate materials and their corresponding surface-functionalization strategies based on the specific requirements of each application.

### 2.3. Nanoparticles

Nanoparticles (NPs) are usually used as a key component for biosensors, such as magnetic nanoparticles (MNPs), AuNPs, and QDs. Surface functionalization is required for these NPs to capture target analytes and facilitate signal generation.

MNP is a material traditionally applied in biosensing. In practical applications, MNPs are usually surface modified with carboxyl groups or SA/biotin for the enabling of connection with antibodies through the treatment of EDC-NHS or by forming SA–biotin bonds. This method has been used in fabricating MNPs for surface plasmonic resonance-based sensing of a hormone [13], detection of oligo and protein interactions [14], and detection of DNA based on magnetic sensors [15].

**Figure 4:**
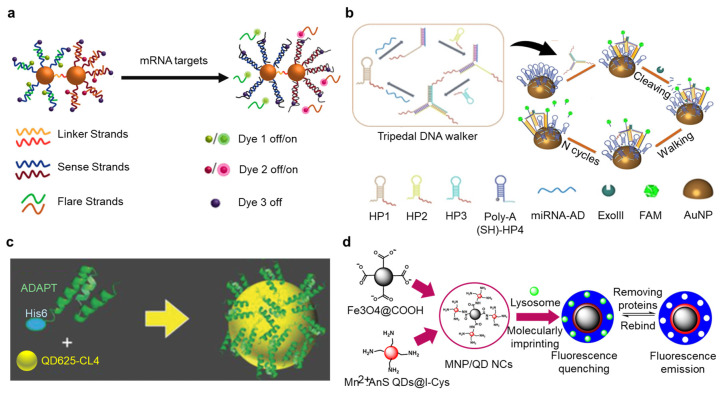
Functionalization of nanoparticles for the capturing and detection of target analytes. (**a**) A nano sensing system capable of detecting two mRNA targets at the same time. The nanoparticle dimer with fluorescent dyes was established by immobilizing thiolated single-strand DNA oligos on gold nanoparticles though gold–thiol bonds. Reproduced with permission from Ref. [17]. Copyright 2018, American Chemistry Society. (**b**) PolyA hairpin DNA molecules labelled by FAM are immobilized on gold nanoparticles through PolyA-mediated binding. Reproduced with permission from Ref. [25]. Copyright 2023, Wiley. (**c**) (His)_6_ motif mediates binding of protein-based probes to surface of Zn-rich quantum dots (QDs) for detection of protein biomarkers based on Förster resonance energy transfer (FRET). Reproduced with permission from Ref. [97]. Copyright 2018, Wiley. (**d**) Molecularly imprinted polymer coated on Fe_3_O_4_ magnetic nanoparticles/*l*-cysteine-modified zinc sulfide QDs for detection of lysosomes based on fluorescent quenching. Reproduced with permission from Ref. [99]. Copyright 2021, Multidisciplinary Digital Publishing Institute.

Functionalization of AuNPs is largely similar to the functionalization of gold electrodes, such as the establishment gold–thiol bonds [16,17] (Figure 4a). However, there are also some special methods for fabrication of AuNPs. For example, binding nucleic acid probes to AuNPs by polyadenine (polyA) is more efficient than building gold–thiol bonds, due to the improved hybridization efficiency and reduced unwanted bond formation [18]. Moreover, a major advantage of polyA-mediated binding is its significantly lower cost in the synthesis of oligonucleotide probes, since there is no need to conjugate extra chemical groups. This strategy can be seen in SERS sensors used to detect multiplexed protein biomarkers of Alzheimer’s Disease [18], fluorescence-based detection of proteins and miRNAs [19,25] (Figure 4b), and fabrication of NPs for test strips based on lateral flow [20].

Apart from MNPs, AuNPs, and composite nanoparticles with gold on their surfaces, there are also other particles that are frequently used in biosensors. QDs are semiconductor nanoparticles with special electronic and optical properties, such as sharp absorption and emission spectra, and the capability to tune their band gap by controlling their size [91]. Therefore, QDs are usually used in optical or fluorescence-based biosensors. Also due to their special fluorescent emission properties, QDs are suitable for being used in multiplexed detection [24]. A common strategy for functionalizing QDs is adding active chemical groups, such as carboxyl or amino groups, directly to QDs during the process of synthesis. For example, diethylenetriamine can be used to synthesize amine-functionalized graphene QDs, and graphene oxide can be used to fabricate graphene QDs with carboxyl groups [92,93]. These QDs can be easily bonded to probes like antibodies or aptamers through the chemical bonds formed between amine group (-NH2) and carboxyl group (-COOH) mediated by EDC-NHS treatment [92,93]. Another approach is decorating QDs with amphiphilic polymers that display carboxyl groups, which can also help antibodies to bind to QDs through the EDC-NHS treatment [94,95]. EDC-NHS can also mediate the binding of antibodies to QDs with hydrophilic thiol groups [96].

The interaction between -COOH and -NH_2_ is commonly employed for functionalizing QDs with biological probes. However, certain non-covalent binding methods offer greater convenience. For instance, polyhistidine peptide tags (his-tags) exhibit a strong affinity for metals, facilitating the attachment of probes to QDs [24,97] (Figure 4c). This approach is advantageous due to its straightforward synthesis and high thermal and enzymatic stability, making it ideal for the conjugation of biological probes with tailored sequences to QDs. Some studies have extensively utilized his-tags in the development of QD-based biosensors employing Förster resonance energy transfer (FRET), enabling the multiplexed detection of nucleic acids and proteins [24,42,97,98]. Moreover, surface modifying techniques that are frequently used in electrodes can also be applied to QDs, such as MIP for capturing target molecules [99] (Figure 4d).

Multiple types of nanoparticles can be integrated into one sensing system to utilize their respective advantages. He et al. designed a sensing system containing CoFe_2_O_4_ nanoparticles, ZnO nanoparticles, and AuAg polyhedron [100]. In this work, ZnO and CoFe_2_O_4_ nanoparticles were aminated by 3-Aminopropyltrimethoxysilane for the surface conjugation with oligos that can specifically bind to target miRNAs. If the target miRNA were to exist, it would form a complex with the ZnO and CoFe_2_O_4_ nanoparticles. ZnO nanoparticles would then be dissolved by HCl to supply Zn^2+^ ions for the triggering of the DNAzyme cycle that cuts S5 oligo to S2 analogues, which can bind to the oligo-decorated AuAg polyhedrons that were immobilized on the sensing layer. The AuAg polyhedron serves as the signal enhancer for SERS detection, while magnetic CoFe_2_O_4_ nanoparticles would be removed by simple magnetic separation [100]. This study is a good example of nanoparticles’ systemic integration, which is also highly suitable for multiplexing, due to the involvement of unique oligos for biomarker capturing.

## 3. Nanomaterials for Improving Sensor Performance

In many past studies related to biosensors, nanomaterials are used to assist the improvement in the sensors’ performance. For instance, certain nanomaterials can enhance the density of sites available for immobilizing probes. Additionally, some nanomaterials can introduce chemical groups that facilitate probe binding to surfaces lacking bioactive functionalities. Furthermore, the unique affinity of specific nanomaterials for certain analytes, as well as the special properties of certain nanomaterials, can significantly improve the detection and quantification of biomolecules.

### 3.1. Polymers

Polymers are highly useful in facilitating the binding of probes to sensors and improving the sensing efficiency. They usually present special geometric, chemical, and bioactive features which can support efficient immobilization of sensing probes and enable sensitive biosensing.

A well-validated form of polymer for biosensing is dendrimer. Dendrimers are nanostructures with a tree-like monodisperse architecture, typically featuring a symmetric core, an inner shell, and an outer shell. Its superior aspects include providing high-density sites for the conjugation of sensing probes and providing spatial separation in order to avoid the aggregation of nanoparticles.

**Figure 5:**
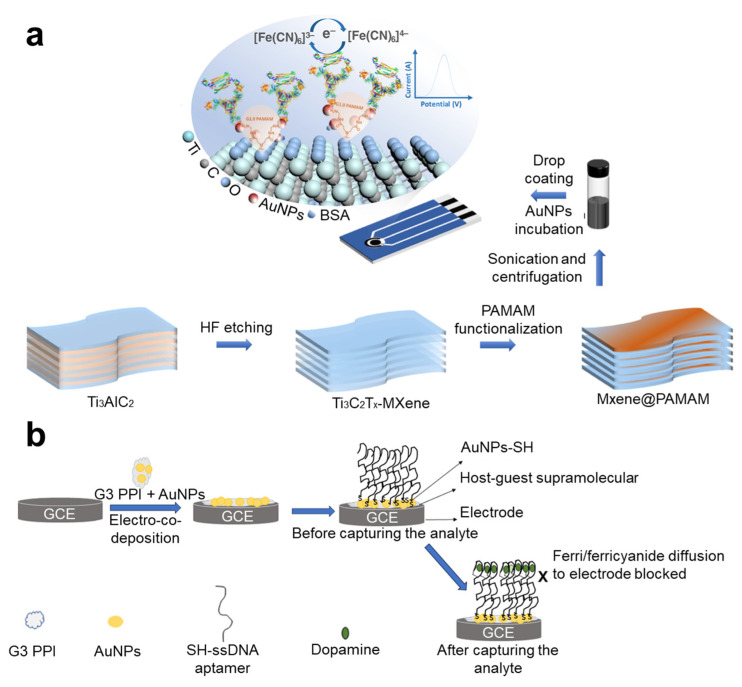
Integrating polymers in biosensors to improve sensing performance. (**a**) Functionalizing MXene with PAMAM dendrimers to improve its electrochemical performance and providing active sites for binding with gold nanoparticles. Hydroxyl groups introduced by HF etching on MXene can react with succinic anhydride, thereby forming active carboxyl terminals for the binding or in situ growth of PAMAM dendrimers. Reproduced with permission from Ref. [23]. Copyright 2022, Springer Nature. (**b**) Functionalizing glassy carbon electrodes with poly(propylene imine) dendrimers and gold nanoparticles by electro-co-deposition for improved immobilization of aptamers. Reproduced with permission from Ref. [54]. Copyright 2023, American Chemistry Society.

One material typically used to generate dendrimers is polyamidoamine (PAMAM). There are in total around 10 generations of PAMAM dendrimers. The generations lower than G4 mainly evince planar structures, low steric hindrance, and high polarity [101]. In contrast, the higher generations (G5 to G10) are increasingly spherical, have higher steric hindrance, and evince a larger cavity in their size [101]. Due to the presence of amino groups at the terminals of its branches, PAMAM can be flexibly applied to functionalize different materials. For example, PAMAM dendrimers lower than G4 can easily be connected to AuNPs through the high affinity between an amino group and AuNPs [23,102,103] (Figure 5a). In the fabrication of electrochemical biosensors, PAMAM dendrimers are usually used to provide binding sites for immobilizing probes, awhile minimizing undesired adsorption [104,105,106]. They can be easily functionalized to electrodes through direct binding to carboxylated surface by EDC-NHS treatment [104], or by binding with other carboxylated nanomaterials like carbon nanotube or graphene oxide for drop-coating onto electrodes [105,106]. In a past study, PAMAM dendrimer was even applied in spatial omics. Cao et al. conjugated PAMAM dendrimers to glass slides silanized with 3-Glycidyloxypropyltrimethoxysilane through creating chemical bonds by reactions between PAMAM’s amine terminal and the epoxy rings of 3-Glycidyloxypropyltrimethoxysilane [107]. The glass slides modified with PAMAM dendrimers can be easily functionalized with amine-conjugated oligonucleotides in order to efficiently capture RNAs in tissue sections, thereby supporting downstream RNA sequencing [107].

Apart from the amine ends, there are also different types of cores for PAMAM which might be utilized for surface functionalization. For example, PAMAM with cystamine core can be processed by sodium borohydride (NaBH_4_) to reduce the core disulfide bonds, and thereby expose active thiol groups to assist in its attachment to a gold electrode surface for electrochemical sensing [108]. PAMAM dendrimers can also be applied as the spatial support for synthesis of other nanoparticles like QDs, and functionalized by antibody for multiplexed detection of proteins based on anodic stripping voltammetric analysis [109].

Other than PAMAM, some other types of dendrimers have also been applied in fabricating biosensors. Sipuka et al. applied electro-co-deposition to functionalize glassy carbon electrodes with generation-3 (G3) poly(propylene imine) (PPI) dendrimers and AuNPs. PPI dendrimers provide good supports for the immobilization of AuNPs, a finding which was further functionalized with thiol-conjugated aptamers for electrochemical sensing [54] (Figure 5b).

Apart from forming dendrimers, some polymers can also serve as the linker between a sensor base and bioactive probes. Poly-L-lysine has been used to bridge the connection between graphene and DNA probes for the detection of multiple types of miRNAs [110]. Poly-L-lysine can also form dendrimers, but their dendrimers are more frequently used in nanomedicine rather than biosensing [111,112]. Because of their high designability, some customized polymers have been proposed to provide multiple types of surface reactive groups for functionalization [113]. Polyethylenimine is also occasionally applied to the surface of electrodes or nanoparticles [114]. Polyethylenimine can bind to biological probes through many ways, including covalent binding through its amino groups [115,116], or by its affinity to biomolecules such as DNA due to electrostatic interactions [117].

### 3.2. Composite Nanomaterials

Similar to polymers, some composite nanomaterials can also be used to functionalize sensor surfaces to assist in the immobilization of biological probes. More importantly, due to the combination of different materials, they can provide multiple benefits by exerting advantages on each component’s respective properties.

One advantage of the functionalization of sensors with composite nanomaterials is the ability to provide chemical groups that facilitate binding of probes. For example, coatings composed of BSA and reduced graphene oxide (rGO) nanoflakes (terminated with amine group) crosslinked with glutaraldehyde have been applied to functionalize gold electrodes to prevent biofouling, improve sensors’ electrochemical properties, and allow the binding of antibodies and other probes for multiplexed biomarker detection [8].

Some composite nanomaterials may exert special properties in assisting biosensing. For example, zinc oxide (ZnO) has a high isoelectric point, which could improve the detection of multiple types of analytes by attracting them to the sensor surface through electrostatic interactions. Nanocomposite formed by ZnO and carbon nano-onion has been deposited to the surface of a glassy carbon electrode for enzyme-less detection of glucose [118]. Cupric oxide has catalytic activities; its nanocomposite with graphene is therefore applied to a carbon paste electrode for the detection of multiple medical molecules [119]. Some carbon-based nanomaterials such as multiwalled carbon nanotube (MWCNT) can also be used to assist functionalization. Periasamy et al. developed a biosensor for detecting glucose by coating gelatin-MWCNT on a glassy carbon electrode to assist the immobilization of glucose oxidase [120]. Polyethyleneimine-wrapped MWCNT has been used to screen a printed electrode for immobilization of antibodies to detect a carcinoembryonic antigen in multiple types of body fluids [121].

**Figure 6:**
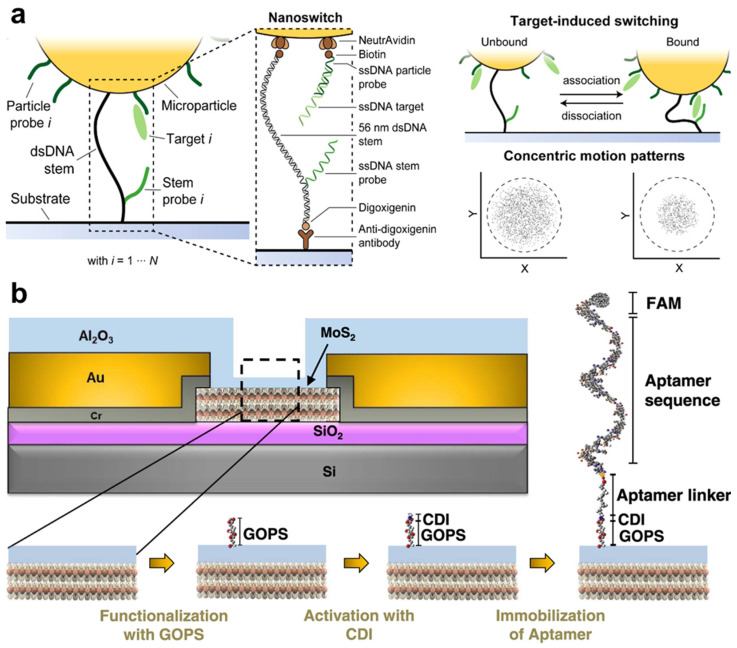
Composite nanomaterials that improve sensors’ performance in multiplexity and sensitivity. (**a**) DNA nanoswitches connecting microbeads to glass substrate, supporting multiplexed detection of target binding. Reproduced with permission from Ref. [22]. Copyright 2020, American Chemistry Society. (**b**) MoS_2_ diode-based field effect transistor containing nanolayers of MoS_2_ and Al_2_O_3_ for ultrasensitive detection of TNF-α in body fluids. Reproduced with permission from Ref. [122]. Copyright 2022, Springer Nature.

One example of a sensing technique that extensively employs composite nanomaterials is the field effect transistor (FET). Composite 2D nanomaterials that have semiconductive properties are expected to foster the development of novel sensitive FET-based biosensors. One prominent example is shown by transition metal dichalcogenides, such as molybdenum disulfide (MoS_2_). De Silva et al. developed a FET-based biosensor capable of detecting femtomole-level concentrations of tumor necrosis factor-alpha (TNF-α) using two-dimensional MoS2 diodes [122] (Figure 6b). In their study, the FET was constructed on a silicon substrate covered with a 300 nm thick layer of silicon dioxide (SiO_2_). The MoS_2_ layer, with a thickness of less than 100 nm, was mechanically exfoliated and placed onto the Si/SiO_2_ substrate. An aluminum oxide (Al_2_O_3_) nano-layer was then deposited onto the MoS_2_ layer for passivation and to facilitate the functionalization of amine-conjugated aptamers. Glycidyloxypropyltrimethoxysilane and 1,1′-carbonyldiimidazole were employed to modify the Al_2_O_3_ surface, enabling the binding of amine group-modified probes [122] (Figure 6b). In addition to MoS_2_, FETs incorporating rGO on SiO_2_ layers have also been reported. In these devices, the rGO surface was modified with 1-pyrenebutyric acid N-hydroxysuccinimide ester (PBASE) through π–π stacking interactions between the pyrene group and rGO. The PBASE modification introduces succinimidyl ester groups that facilitate binding with amine groups, thereby enabling effective surface functionalization [123]. Multiplexing capabilities can be achieved using FET arrays, where different FETs are functionalized with distinct probes [123]. One significant advantage of multiplexed FET-based biosensors is their high specificity, which arises from the unique doping effects induced by target analytes with varying isoelectric points, which results in different shifts in the transfer characteristic curves [123].

Composite nanomaterials also enable innovative sensing modes. Nucleic acids are highly versatile and exhibit strong affinity for various biomolecules. Lubken et al. developed a material called the nanoswitch, based on DNA strands, which serves as a linker that binds to a microparticle and immobilizes it on a glass slide (Figure 6a). The dynamic binding of analytes leads to deformation of the nanoswitch and displacement of the microparticle, which can be quantified through image analysis [22] (Figure 6a). Importantly, the nanoswitch linkers bind reversibly to target analytes, with the association and dissociation kinetics determined by different sequences of the nanoswitch and their target analytes. This variation in binding kinetics is reflected in the concentric motion patterns of each individual microparticle (Figure 6a), which can be recorded, quantified, and analyzed through imaging techniques. Hundreds of these nanoswitch-bound microparticles, each targeting different analytes, can be monitored simultaneously, with their target molecules decoded through their unique “motional barcodes”, allowing for the estimation of the concentrations of multiple analytes.

Composite nanoparticles themselves can also be applied to modify surfaces of biosensors to achieve desired properties. Nie et al. developed TiO_2_@Pt nanoparticles with a hairpin-shaped nucleic acid probe functionalized on its surface through a Pt-S bond, and deposited the nanoparticles on a glassy carbon electrode to utilize the material’s property of promoting electron transfer, which was demonstrated on multiple types of miRNA biomarkers [56].

In summary, employing composite nanomaterials for the functionalization of biosensors offers flexible strategies to leverage the synergistic properties of different materials, thereby improving their performance in various aspects. The continuous discovery of innovative composite nanomaterials holds the potential to further elevate the efficacy of biosensing technologies.

## 4. Paradigms for Multiplexed Molecular Biosensing

Multiplexed detection of biological analytes is a major direction for the development of novel biosensing methods. Despite the similarity in the principle of functionalization compared with single-analyte sensors, the fabrication of multiplexed biosensors requires some special strategies to allow for the simultaneous detection of multiple biomarkers. In general, there are three different approaches to achieve the goal of multiplexed sensing, and they require different strategies in surface functionalization.

### 4.1. Spatial Separation of Detecting Regions

**Figure 7:**
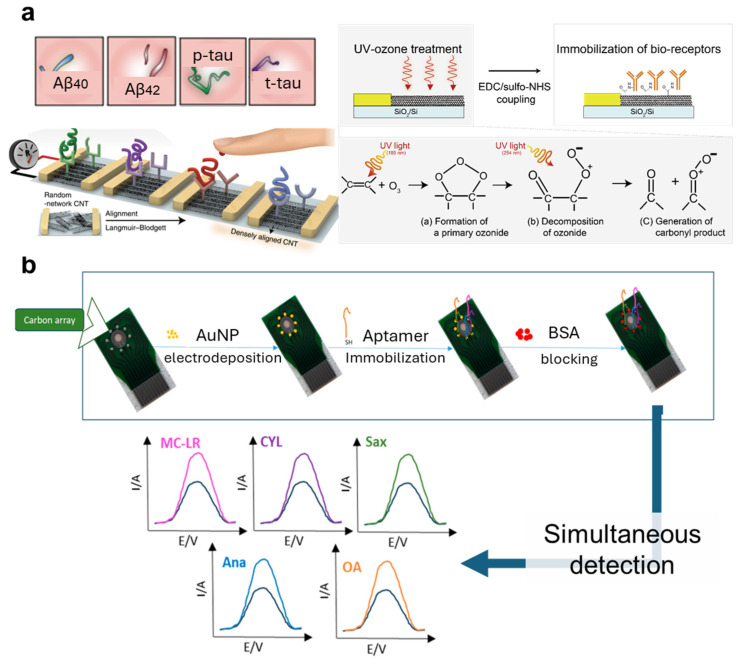
Multiplexed sensors based on spatially separating sensing regions that are functionalized with different probes. (**a**) Spatially separated functionalization of carbon nanotubes for multiplexed detection of biomarkers related to Alzheimer’s disease. Ultraviolet–ozone was used to generate carbonyl groups on carbon nanotubes, followed by EDC-NHS treatment to activate the surface, and the dropping of antibodies to predesigned sensor regions. Reproduced with permission from Ref. [26]. Copyright 2020, Springer Nature. (**b**) Multiplexed electrochemical aptasensor for detection of multi-toxin. Spatially separated electrodes with deposited gold nanoparticles were incubated with their respective thiolated aptamers for functionalization. The aptamers form self-assembled monolayers that improve sensor stability. Reproduced with permission from Ref. [131]. Copyright 2024, Multidisciplinary Digital Publishing Institute.

Spatial separation is the simplest way to achieve multiplexed detection. For example, many studies based on lateral-flow devices have applied jetting of probes directly onto different regions of test strips to achieve detection of multiple biomarkers in a single test [124,125,126]. Similarly, the functionalization of electrical, optical, and fluorescence-based biosensors that achieve multiplexing through spatial separation can also be performed by separately applying different probes to their corresponding sensing regions [8,26,48,127] (Figure 7a). To achieve good outcomes of functionalization, techniques that support the precise coating of reagents to specific regions of biosensors are sometimes adopted, especially nanofabricating methods such as inkjet printing or spotting [127,128].

Some microfluidics-based technologies have also adopted a special method that applies the same surface functionalization to the whole microchannel, with molecules that capture signaling probes (such as avidin for biotin-labeled probes), while the multiplexed detection is achieved by dividing the sample liquid into multiple, distinct portions and distributing them to different regions of the channels for quantification [129]. The key to this strategy is functionalizing the sensing device with a universal capturing probe, while dividing the sensing reactions into spatially separated regions, which is more flexible as to various applications, since the same device can be applied for different tasks with different target analytes.

In summary, all kinds of common functionalizing strategies can be used for establishing multiplexed biosensors based on the spatial separation of their detecting regions. Notably, some methods that reduce non-specific binding and improve sensor stability might be required for this type of multiplexed biosensors, such as the coating of nanolayers on electrodes [130,131] (Figure 7b).

### 4.2. Unique Identifiers

One effective way to detect multiple biomarkers from a limited amount of sample is to apply unique identifiers for classification. Multiple types of identifiers have been used in past studies, mostly related to the application of materials that generate different signals.

Many biosensors based on nanoparticles apply the strategy of unique identifiers in order to achieve multiplexing. Raman spectroscopy assisted by nanostructures used to enhance signals is capable of detecting multiple analytes based on their unique Raman spectra [132], especially organic molecules. However, Raman’s distinguishability towards macromolecules like proteins is relatively low, limiting its applications in this area. SERS tags with target-specific probes can overcome this problem. By functionalizing probes targeting different biomarkers on different Raman dye-coded nanoconjugates, the varied levels of different biomarkers can be easily realized [18,133]. QDs that generate distinct electrical, optical, and fluorescent signals are also commonly employed to achieve multiplexing. This is accomplished by functionalizing the QDs with various probes and mixing them in a single reaction system, where different signal tags are used to target specific analytes [109,134,135]. DNA barcodes are also useful agents, since they are easy to amplify and flexible to customize. Nanoparticles decorated with different antibodies and DNA barcodes have been applied along with nanopore sensing for multiplexed detection of cancer biomarkers [136].

**Figure 8:**
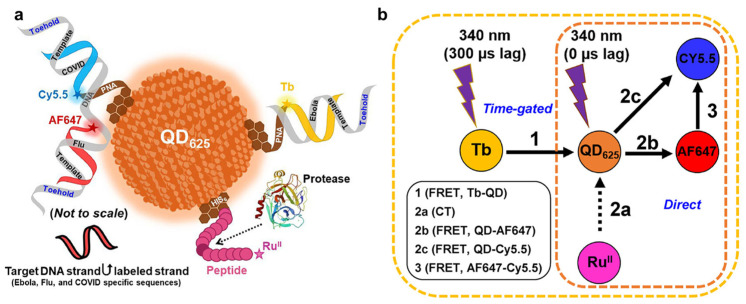
Multiplexed detection of DNAs and proteases by QDs. (**a**) Capturing probes based on oligo nucleotides and peptides were immobilized on the same QD. (**b**) Multiplexing of the QD-based biosensor was achieved by FRET. Reproduced with permission from Ref. [24]. Copyright 2024, American Chemistry Society.

Advancements in nanomaterials and in their integration with oligonucleotides have led to the development of novel multiplexing techniques. For instance, the nanoswitch and microparticle sensing system proposed by Lubken et al. enables the detection of various analytes by quantifying their binding periods, which indicates their different levels of affinity to the nanoswitch [22]. Similarly, Hastman et al. modified QDs with oligonucleotides hybridized to complementary strands labeled with dyes, as well as peptides modified with electroactive groups (Figure 8a). This system can detect multiple target DNAs and proteins through changes in fluorescence emission spectra resulting from FRET [24] (Figure 8b). These methods demonstrate that nanomaterials capable of generating multiple types of signals have significant potential for multiplexed biosensing, offering advantages in the simplification of detection systems while enhancing multiplexing capabilities. Furthermore, this insight emphasizes the technologies that could drive the future development of multiplexed sensors, such as molecular fluorescent multicolor indicators, which can generate distinct fluorescent signals based on different conformational states or interactions with specific analytes [137].

In general, biosensors that achieve multiplexed detection by unique identifiers normally do not require special surface-functionalizing methods. Instead, the selection of signal-generating agents is important. Functionalization is usually performed separately on different signal-generating agents to afford different targeting capabilities, and these signal-generating agents would be mixed for multiplexed detection. Novel nanomaterials with different levels of quantifiable reactions to different analytes also have huge potential with respect to their use in the making of multiplexed biosensors.

### 4.3. Multimodal Detection

In many biosensing tasks, the target biomarkers can be largely different in their forms and properties. Under this situation, multimodal sensing systems would be required. One major problem to be considered under this situation is how to detect different signals simultaneously, without interference between each other.

**Figure 9:**
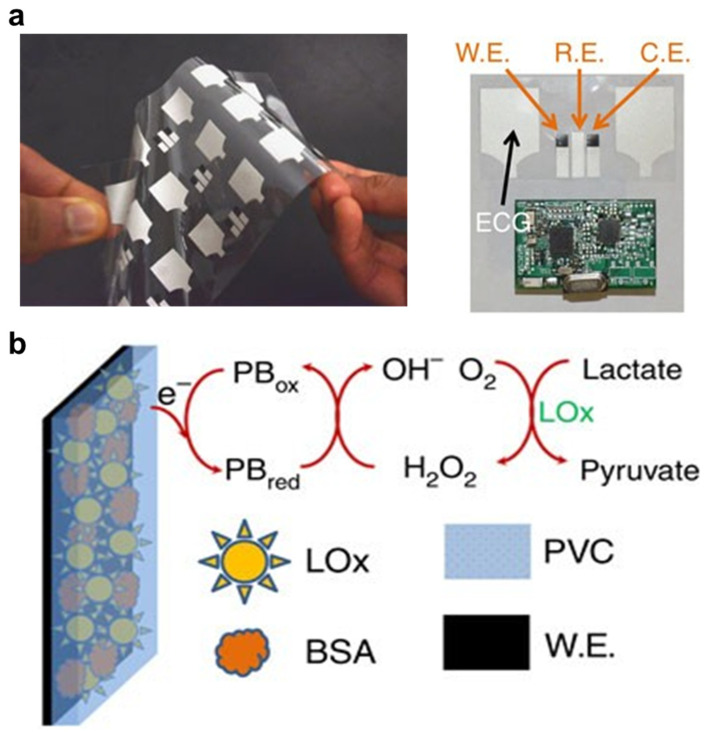
Multiplexed biosensor based on multimodal signal detection. (**a**) Flexible multimodal patch for simultaneously measuring ECG and lactate. (**b**) Electrodes of the sensor were fabricated by sequential screen printing of conductive material, oxidase-modified Prussian blue, and insulator (PVC: Polyvinyl chloride) inks. Reproduced with permission from Ref. [115]. Copyright 2016, Springer Nature.

One effective way to perform multimodal biosensing is integrating multiple sensing elements together in a system, while keeping them separated. For example, Zahed et al. designed a wearable patch for monitoring multiple physiological biomarkers, including glucose, pH, skin impedance, and electrocardiography [50]. The patch contains MXene–polyvinylidene fluoride nanofiber for the detection of forces, polyaniline-based nanofibrous porous layer to measure pH, and gold electrode coated with nanolayer of rGO for the measurement of glucose (by glucose oxidase immobilized on a carboxylated rGO layer through EDC-NHS treatment) [50]. In some cases, to further avoid interference between electrode areas used for different purposes, insulators can be added to seal and separate different sensing regions. Imani et al. designed a multimodal patch to measure ECG and lactate in human sweat (Figure 9a), which applied screen printer to sequentially print Ag/AgCl (conductive material for working and counter electrodes) on electrodes for the detection of both ECG and lactate, and further print lactate oxidase-modified Prussian blue as well as insulator inks for functionalizing the lactate-sensing electrodes [138] (Figure 9b).

Advancements in materials may also provide novel ideas for multimodal biosensing. A recent study proposed a conductive hydrogel based on modified poly(3,4-ethylenedioxythiophene):poly(styrenesulfonate) and gelatin. This hydrogel provides responsive signals related to urea, body temperature, and even cardiac conditions [139]. These types of multimodal materials provide novel ideas for the functionalization of biosensors for achieving multiplexed sensing. We expect that multimodal composite nanomaterials might also have great potential to be applied in such a kind of multiplexed biosensor. In fact, multimodal nanomaterials have already been applied in multiplexed detection of biomolecules; Tort et al. developed a DNA-gold nanoparticle for the detection of biomarkers based on localized SPR and spatial information for a particle binding to a detecting slide [140]. Although multimodal detection in this study was used for improving detectability rather than directly providing quantification of different biomarkers, this technique still provides a good reference for future study in this area.

In summary, strategies for multiplexed biosensing can be roughly divided into two paradigms. Currently, most of the multimodal biosensors integrate sensor elements functionalized for different types of biomarkers together into one sensor. However, with the development of novel composite materials, we can expect that materials with multimodal properties might be used more and more in functionalizing and developing biosensors.

## 5. Summary and Outlook

Biosensors are continuously infiltrating into people’s lives, providing support for diagnosis, daily healthcare management, chemical monitoring, food safety, and biomedical research. The application scenarios are becoming more and more diverse, along with higher requirements for precision and comprehensiveness of detection. These requirements are leading to several trends in biosensor development. Firstly, the awareness of the importance of multiplexing has been raised, especially for solving complex problems, such as diagnosing heterogeneous diseases and point-of-care testing [7,11,18,110]. Secondly, aiming at the tackling of tasks related to different types of samples, analytes, and scenarios, novel biosensors are evolving to have diverse structural features and sensing principles. Thirdly, composite materials are commonly used more and more in biosensors to overcome the limitations of single materials.

The trends in biosensor development are leading to the widespread adoption of nanomaterials and nanofabricating technologies. Nanomaterials and nanostructures have many advantages that can hardly be gained otherwise. For example, nanostructures like SAMs [9,32,33,39,71,74,76], MIPs [10,11,12], and dendrimers [23,54,102,103,107,108,109] provide ideal spatial and chemical supports for the binding of biological probes, or even the direct capture of biomolecules. Certain types of nanoparticles are widely used as tags for the generation or enhancement of quantifiable signals, such as MNPs [13,14,15], AuNPs [16,17,18,19,20], QDs [91,92,93,94,95,96], and composite nanomaterials that contain these nanoparticles as core elements [23,54,60,100]. Some 3D nanostructures on certain materials (such as gold) have special optical effects that can be utilized by biosensors based on SERS and SPR [66,67,68,69]. Therefore, advancements in nanomaterials and nanofabrication have become major promoters for the development of novel biosensors.

We can clearly see that nanotechnologies are fostering new modes of biosensors, especially multiplexed detections. Traditionally, multiplexed biosensors based on the spatial separation of detecting regions apply nanomaterials merely for surface modification and electrode fabrication to improve their performance (e.g., SAM) [8,26,130,131], or apply basic nanoparticles as tags to denote the existence of analytes (e.g., lateral flow) [124,125,126,127]. However, nanomaterials have much more potential in supporting multiplexed detection based on more integrated manners of use. Due to their high flexibility in customizing structures, nanoparticles can be fabricated to carry different signal-generating components and molecule-capturing probes, which are highly useful in multiplexed biosensors based on unique identifiers [18,109,133,134,135]. In recent years, the involvement of oligo nucleotides as a part of a nano sensing system also further extended the forms and capabilities of identifiers for multiplexing [22,136]. With the development of novel nanomaterials and nano-fabricating technologies, new patterns of biosensing are expected to continually appear.

The bridge that links nanotechnologies with biosensing capability is surface functionalization. Surface functionalization for biosensors includes two major types. The first type is the functionalization of nanomaterials for the carrying of biological probes. This method is commonly seen in biosensors based on nanoparticles with signal-generating capabilities. Most of these nanoparticles are immobilized with probes based on covalent bonds such as SA–biotin bonds, the gold–thiol bond, and the peptide bond [13,14,15,16,17,18,19,20,23,54,60,91,92,93,94,95,96,100], while a few of them adopt simpler non-covalent bindings that utilize affinities between certain motifs and nanoparticles [18,19,20,24]. The second type of functionalization is the use of nanomaterials to modify sensing surfaces and improve sensors’ performance. Several types of SAMs, MIPs, dendrimers, and nanoparticles are commonly used in the modification of electrodes for electrochemical biosensors, for the purpose of facilitating immobilization of bioactive probes, protecting electrodes from contamination, exerting catalytic effects, and modulating electron transfer [8,9,10,11,12,22,32,33,39,56,71,74,76,104,105,106,118,119,120,121]. Importantly, although the methods for surface functionalization are very diverse, most of them are based on several basic chemical reactions. Although this review cannot cover all the recent progress related to the application of nanotechnologies in biosensing, we have reviewed the major types of strategies for surface functionalization, with representative examples provided.

In the near future, we anticipate that multiplexing capabilities will be a key consideration in the development of new biosensing systems, with nanomaterials and nanofabrication serving as the foundations for innovations in this field. Researchers can contribute to the advancement of novel biosensors through several approaches. Firstly, in sensor fabrication, there is a pressing need for innovative materials for sensor bodies that guarantee stable, reproducible, and high-efficiency performance. Composite nanomaterials which offer stability, high affinity for biological probes, and excellent electrical properties are particularly promising in this regard. Secondly, in application design, researchers should focus on creating sensors that excel in challenging yet critical tasks for biological research, such as sampling biomarkers from deep tissues or within the cytoplasm by the application of 3D structures or multiplexed capturing probes. Thirdly, the exploration of novel nanomaterials with favorable properties for surface modification or those that possess multiplexed sensing capabilities is highly valuable. Moreover, significant innovations are required in the system integration process to establish functional sensing systems, particularly in surface-functionalization methods that effectively bind biological probes to sensors and support the required sensing tasks. By summarizing the surface-functionalization strategies related to biosensors, this review aims to serve as a reference supporting interdisciplinary technical integrations and as a foundational guideline for the initiation of research in this area.
